# Landscape of Innovative Methods for Early Diagnosis of Gastric Cancer: A Systematic Review

**DOI:** 10.3390/diagnostics13243608

**Published:** 2023-12-05

**Authors:** Alexandra Orășeanu, Mihaela Cristina Brisc, Octavian Adrian Maghiar, Horia Popa, Ciprian Mihai Brisc, Sabina Florina Șolea, Teodor Andrei Maghiar, Ciprian Brisc

**Affiliations:** 1 Clinic of Gastroenterology, Bihor Clinical County Emergency Hospital, 410169 Oradea, Romania; adaoraseanu@yahoo.com (A.O.); sabi_florina95@yahoo.com (S.F.Ș.); 2 Doctoral School of Biomedical Sciences, University of Oradea, 410087 Oradea, Romania; octimaghiar@gmail.com (O.A.M.); teodormaghiar@yahoo.com (T.A.M.); brisciprian@gmail.com (C.B.); 3 Faculty of Medicine and Pharmacy, University of Oradea, 410068 Oradea, Romania; brisciprian08@gmail.com; 4 Clinical Emergency Hospital “Prof. Dr. Agrippa Ionescu”, 011356 Bucharest, Romania; horia.popa09@gmail.com

**Keywords:** gastric cancer, metabolomics, biomarkers, endoscopy, gastroenterology, screening

## Abstract

From a global perspective, gastric cancer (GC) persists as a significant healthcare issue. In the Western world, the majority of cases are discovered at late stages, when the treatment is generally unsuccessful. There are no organized screening programs outside of Asia (Japan and Republic of Korea). Traditional diagnosis techniques (such as upper endoscopy), conventional tumor markers (CEA, CA19-9, and CA72-4), radiographic imaging, and CT scanning all have drawbacks. The gold standard for the earliest detection of cancer and related premalignant lesions is still endoscopy with a proper biopsy follow-up. Since there are currently no clinically approved biomarkers for the early diagnosis of GC, the identification of non-invasive biomarkers is expected to help improve the prognosis and survival rate of these patients. The search for new screening biomarkers is currently underway. These include genetic biomarkers, such as circulating tumor cells, microRNAs, and exosomes, as well as metabolic biomarkers obtained from biofluids. Meanwhile, cutting-edge high-resolution endoscopic technologies are demonstrating promising outcomes in the visual diagnosis of mucosal lesions with the aid of linked color imaging and machine learning models. Following the PRISMA guidelines, this study examined the articles in databases such as PubMed, resulting in 167 included articles. This review discusses the currently available and emerging methods for diagnosing GC early on, as well as new developments in the endoscopic detection of early lesions of the stomach.

## 1. Introduction

Globally, 1.2 million people are diagnosed with GC each year, and 860,000 people die from it [1], making it the fifth-leading cancer diagnosis and the third-leading cause of cancer mortality [2]. The majority of the world’s GC outcomes remain poor, including in the United States (US). GC affects 27,000 people in the United States each year [3] and has a dismal prognosis (5-year survival rate of 27%) [4]. These statistics reflect the fact that the vast majority of GCs in the United States are diagnosed at advanced stages [4], making curative resection unlikely. Strategies to improve the early detection of GC are thus critical to improving survival.

There are notable regional variations in the onset of GC, with East Asian nations like Japan and Korea being known to have a particularly high incidence rate when compared to Western nations. GC is now more frequently found in the asymptomatic stages in Japan as a result of the implementation of a mass screening program that uses double-contrast barium radiography for the early detection of the disease, as well as advancements in endoscopic equipment and improved diagnostic accuracy [5]. As a result, roughly 50% of cases of GC that are currently being treated in Japan are in their early stages [6].

In contrast, GC is frequently discovered at an advanced stage and the prognosis is still poor in Western nations. The only option for treating cancer is complete excision, and the prognosis is dependent on the stage at which it is discovered. It is crucial to find the cancer at the earliest stage possible, as evidenced by the excellent postoperative outcomes for early gastric cancer (EGC), which have a 5-year survival rate of over 90% in both Western nations and Japan [7,8].

The currently available diagnosis methods include common ones such as radiographic imaging and CT scanning, upper endoscopy, and traditional tumor biomarkers (CEA, CA19-9, and CA72-4).

At present, minimally invasive techniques like endoscopic therapy and laparoscopic surgery are used to treat early-stage GC; however, multimodality treatment, such as chemotherapy, radiotherapy, and surgery, is required to treat advanced-stage cancer. In the near future, improving the prognosis of advanced GC is necessary; therefore, as a result, non-invasive diagnostic techniques like genetic and metabolic biomarkers are now used as GC screening instruments.

### Classification

Regardless of lymph node metastasis, EGC can be characterized as remaining comprised to the mucosa or submucosa [9]. The well-known Japanese GC macroscopic classifications categorize EGC into three macroscopic types (0-I, 0-II, and 0-III), with 0-II being further divided into 0-IIa, 0-IIb, and 0-IIc. 0-IIc is the most prevalent subtype of EGC [10]. If there is a mixture of two or more macroscopic types, the type that has propagated the most is recorded first. The Paris classification, which has its foundation in the Japanese classification, was suggested at an international meeting held in 2002 and later accepted as a straightforward and globally standardized classification for superficial gastrointestinal cancers [11].

## 2. Material and Methods

### 2.1. Search Strategy

In accordance with the Preferred Reporting Items for Systematic Reviews and Meta-Analyses (PRISMA) guidelines, a systematic search was conducted using the databases PubMed, Scopus, and Web of Science to find potentially pertinent articles.

The terms “gastric cancer”, “machine learning”, “screening”, “early diagnosis”, and “biomarkers” were paired with the Boolean operators “AND” and “OR” to create an organized search strategy. Only finished research was considered for inclusion.

### 2.2. Eligibility Assessment and Data Extraction

Excluded from consideration were review articles, case studies, editorials, viewpoints, conference abstracts, news articles, and papers that were not written in English. To find more pertinent articles, the reference lists of the chosen articles were screened. Review articles were also obtained in order to find more potential studies.

### 2.3. Outcomes

The main goal of this study was to determine the current and new techniques for EGC diagnosis, along with advancements in the endoscopic detection of early stomach lesions.

Single-arm studies were included, as well as ones with a comparative group (for example, neoplastic lesions of the stomach versus non-neoplastic lesions).

### 2.4. Results

**Figure 1:**
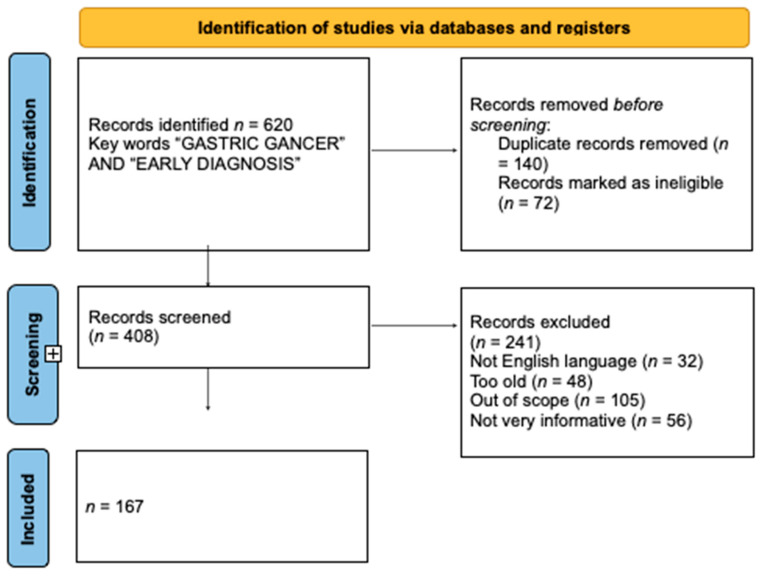
The PRISMA 2020 flow diagram. From Page MJ, McKenzie JE, Bossuyt PM, Boutron I, Hoffmann TC, Mulrow CD, et al. The PRISMA 2020 statement: an updated guideline for reporting systematic reviews. *BMJ* **2021**, *372*, 71. doi:10.1136/bmj.n71. [12].

A total of 620 articles were found in the aforementioned databases after the searches. The samples included in the reviewed studies were geographically distributed across all continents, while most of them were conducted in Asia, and the subjects in the articles were both male and female. The majority of studies tried to differentiate benign lesions from neoplastic lesions. A total of 140 duplicate published trials were eliminated, and 72 ineligible studies were excluded. After a further evaluation of their relevance to this study’s topic, 408 studies remained. A total of 241 articles had to be eliminated in order to select the ones that were the most pertinent to this theme, as mandated by the screening process. A total of 32 were omitted because they were not written in the English language. After that, 48 were eliminated because they were outdated. Furthermore, 105 articles were removed because the summaries were not available or because they were not compatible with our theme. A total of 56 studies were removed because they failed to meet the standards for high-quality data extraction. In the end, the analysis led to the inclusion of 167 references in this review. The algorithm’s results are shown in Figure 1 and in Table 1 study characteristics are presented. Also, the PRISMA 2020 Checklist [12] is available in Supplementary Materials section.

## 3. Classical Diagnosis Methods

### 3.1. Radiographic Diagnosis

Radiographic screening entails the ingestion of a contrast material—often barium—and then the imaging of the gastric lumen fluoroscopically afterward. Ulcers, polyps, and masses can all be found in luminal pathology using contrast radiography. However, radiography has a lower sensitivity and specificity than contemporary endoscopy [22]. According to cancer registry data, radiographic screening has a sensitivity of 60–80% and a specificity of 80–90% [5]. It should be noted that radiography’s sensitivity has been reported to be significantly lower (14–36%), especially for EGC (where luminal prominence or depression may be minimal) [23,24].

Numerous observational studies, including case–control and cohort studies, have evaluated the effectiveness of radiographic screening [25], but it should be noted that no randomized control trial has been carried out to compare radiographic screening with the standard of care. Receiving radiographic screening was linked to lower rates of GC-specific mortality (with a relative risk ranging from 0.52 to 0.54), as well as all-cause mortality, in cohort studies from Japan [26,27] comparing radiographic screening with no screening over a long-term follow-up (ranging from 11 to 13 years) (relative risk of 0.71–0.83).

However, radiography was also a common test used to evaluate gastrointestinal symptoms during the time of these studies, raising the possibility of confounding by indication. Furthermore, it is unknown whether any additional screenings (like endoscopies) were received in the follow-up period, which could have led to an overestimation of the effect size. Radiographic screening has a generally good safety profile, with only a slight chance of constipation or ileus and a very small number of cases of aspiration pneumonia [5].

In settings with limited resources, radiography may still be used for GC screening, as it is a fairly safe and affordable modality. However, compared to contemporary, high-resolution gastrointestinal endoscopy, radiography has limited utility because the main goal of screening is to improve the detection of early-stage cancers. Furthermore, upper endoscopy is needed for tissue collection and visualization after radiography identifies an abnormality.

### 3.2. Computed Tomography Imaging

Another frequently used method to evaluate local tumor invasion is computed tomography imaging (CT). Short scanning times and a simultaneous visualization of the abdomen and thorax are benefits. However, CT offers subpar contrast for soft tissues, necessitates intravenous contrast material and sufficient stomach distention for focused image evaluation, and is always accompanied by radiation exposure [28]. CT and endoscopic ultrasound produce results that are comparable in terms of diagnostic precision for the T staging of GCs. Based on a review of the findings of six studies, the performance of CT for overall T staging revealed a diagnostic accuracy ranging from 77.1 to 88.9%. Sensitivity and specificity ranged from 82.8 to 100% and from 80 to 96.8%, respectively, for determining serosal involvement [28].

However, CT has its own limitations in viewing small, early lesions and has drawbacks that include the requirement of radiation exposure and the use of a contrast agent in most instances, which may make it inappropriate for individuals who have severe kidney issues.

### 3.3. Endoscopic Diagnosis 

The preferred diagnostic imaging procedure for the tissue diagnosis and tumor localization of gastric carcinoma at the moment is esophagogastroduodenoscopy (EGD). When combined with an endoscopic biopsy for tissue diagnosis, EGD is a highly sensitive and specific diagnostic test [29]. Endoscopically, GC usually shows as a friable, ulcerated mass. The existence of nodular folds or thickened irregular margins in patients with endoscopic gastric ulcer findings is also suggestive of the presence of malignancy [29].

Any suspicious-appearing gastric lesions or ulcerations discovered on upper endoscopy should be biopsied because up to 5% of malignant ulcers grossly resemble benign lesions. The sensitivity of a single biopsy in detecting GC is about 70%, and it rises to 98% after seven biopsies along the base and margin of the ulcer [29]. As the early detection of GC offers the best chance for cure and long-term survival, it is crucial to take numerous biopsies from suspicious-looking lesions and smaller, benign-looking gastric ulcers in patients who are at higher risk of the disease.

However, additional diagnostic testing is required when both benign and malignant signs are noticeable or when undefined results are reported. It will take more testing to identify metastases before treatment options can be decided. A helpful technique for identifying liver metastases larger than 5 mm in diameter, perigastric involvement, peritoneal seeding, and the involvement of other peritoneal structures is CT scanning, as mentioned above. CT scanning, however, cannot determine whether a tumor has spread to nearby lymph nodes unless those nodes are enlarged. Furthermore, it has not been demonstrated to be useful in determining the depth of tumor invasion and is not able to consistently facilitate the identification of single liver or lung metastases with a diameter of less than 5 mm [30].

The technology known as endoscopic ultrasonography (EUS) makes staging more precise. High-frequency soundwaves are used in EUS to detect local lymph node involvement, which may be evaluated via an operative biopsy, and to measure the depth of tumor invasion. The transducer is positioned directly next to the gastric wall. Because survival is influenced by tumor depth and lymph node involvement, EUS plays a critical role in improving preoperative staging accuracy. EUS cannot screen for lung or liver metastases or evaluate the involvement of distant lymph nodes because it cannot evaluate tissue below approximately 5 cm in depth. In order to best determine the number and location of involved lymph nodes, the preoperative staging of GC using a combination of endoscopy and CT scanning is supported by the recent literature [31]. 

#### 3.3.1. White Light Endoscopy

Understanding the traits of early-stage disease and becoming proficient in endoscopic observation techniques are essential for making an accurate EGC diagnosis. It is crucial to pay attention to details when performing white light endoscopy, including minor changes in the color of the mucosa (pale redness or the vanishing of color), the loss of visibility of the submucosal vessels underneath, thinned and ruptured mucosal folds, and spontaneous bleeding. The endoscopist must be careful to remove mucus stuck to the gastric wall, suction the gastric juice in excess, supply enough air, and carefully examine the whole region within the stomach because each of these findings is frequently a subtle change. 

In addition, when a lesion is seen, the endoscopist makes a thorough diagnostic assessment taking into account the mural thickness and firmness, color, fold concentration, depression profundity, and protrusion length, even though a diagnosis of invasion depth is necessary. The stomach’s air volume can be reduced to observe the wall’s hardness and thickness. According to reports, conventional endoscopy is really only 72–84% accurate at differentiating between intramucosal and submucosal carcinoma [32,33].

#### 3.3.2. Chromoendoscopy

It has been demonstrated that a careful chromoendoscopic observation can effectively assist diagnosis when subtle mucosal changes like those previously described are seen. In most cases, the forceps channel is used to directly spray 0.1% indigo carmine. The dye must also be liberally sprayed over the mucosa encircling the lesion because EGC is diagnosed by comparing it to the surrounding mucosa. This technique brings out minute variations in the mucosal surface’s elevation, as well as modifications to the surface’s structure and color, which aids in improving both the qualitative diagnosis and the assessment of the degree of invasion [34,35].

However, when mucus is fixed to the stomach wall, indigo carmine spraying can really muddy the lesion boundaries [36]. It is crucial to wash the lesion thoroughly before spraying.

#### 3.3.3. Narrowband Imaging

By illuminating the mucous layer with blue and green narrowband lights, narrowband imaging (NBI), a common type of equipment-based image-enhanced endoscopy, improves the mucous layer’s superficial surface structure and vascular architecture. The gastric mucosa’s microvascular (MV) and microsurface (MS) patterns can be seen in great detail with magnified endoscopy and NBI. The “VS classification” system, developed by Yao et al., merges changes in these patterns with the existence or lack of a demarcation line to diagnose GC [37]. In two key areas, this approach has demonstrated effectiveness in real-world clinical settings.

The first area is the distinction between gastritis and small GC. It has often been challenging to accurately distinguish between minor GC and benign variations (such as gastritis) using traditional endoscopic observation. According to a prospective randomized controlled trial that looked at the differential diagnosis of gastritis and small depressed stomach cancer with depressed lesions less than 1 cm in diameter, combined NBI magnification was more accurate than traditional white light endoscopy [38].

The value of using NBI magnification for margin determination during the complete examination prior to endoscopic therapy is the second area. Although ensuring lateral margins based on a precise diagnosis is important during endoscopic therapy, in about 20% of cases, it is difficult to pinpoint the margins for initial GC using both conventional endoscopy and chromoendoscopy [39,40].

### 3.4. Classic Tumor Markers

Currently, the carcinoembryonic antigen (CEA), carbohydrate antigens (CAs)—CA19-9, CA72-4, CA125, CA24-2, and CA50—and pepsinogen and alpha fetoprotein (AFP) are the tumor markers most frequently used in the clinic for the early detection of GC [41]. None of these serum biomarkers is currently unique for the diagnosis of GC due to their low specificity and sensitivity [41,42]. As a result, it is essential to develop better detection techniques to identify CG (chronic gastritis) in its early stages, especially given that the majority of patients experience no symptoms until the disease has reached an advanced stage. Additionally, GC is a multifactorial, heterogeneous disease with numerous genetic and epigenetic alterations [43].

Recently, new understandings of the molecular pathogenesis have been gained through the use of high-throughput technologies, leading to a new molecular categorization of gastric adenocarcinomas into four subtypes based on their genomic characteristics. The Cancer Genome Atlas (TCGA) categorizes GCs into three categories: chromosomally unstable tumors (CINs), microsatellite instability tumors (MSIs), and Epstein–Barr virus (EBV)-infected tumors (EBV-ITs) [44].

MSI tumors and microsatellite-stable (MSS) tumors with either epithelial-to-mesenchymal transition (MSS/EMT), TP53 activity (MSS/TP53+), or TP53 inactivity (MSS/TP53-) are the two subtypes of GC according to the Asian Cancer Research Group (ACRG) [45,46]. This new classification paved the way for several clinical trials with promising outcomes [47] that sought to define novel therapeutic treatment plans combining immune checkpoint inhibitors with molecularly targeted therapies. Early diagnosis is still necessary, though, and research aimed at finding fresh biomarkers or genetic signatures is crucial.

## 4. Genetic Biomarkers

Surgery and systemic chemotherapy are currently the main treatments for GC patients; radiotherapy, immunotherapy, and targeted therapy are also progressively being used [48], but the 5-year survival rate of gastric malignancy patients is still subpar. As a result, GC treatment and prognosis greatly depend on the early detection of GC [49]. Imaging and pathological biopsy are the two main methods used to diagnose GC [50]. Imaging, however, cannot be used to monitor tumors in real time and exposes patients to radiation, while pathological biopsy is an invasive test that affects patients. Contrarily, a liquid biopsy is becoming more and more accepted as a tool for the real-time monitoring, treatment, and diagnosis of GC [51].

A liquid biopsy can, in some cases, take the place of traditional invasive physical biopsies as a non-invasive detection technique. Despite all of its benefits, there are some restrictions and challenges in its analysis. For those with GC who are at a high risk of negative results, a new method with high sensitivity is therefore required. Liquid biopsy studies in the area of cancer have increased in recent years as a result of several articles’ thorough summaries of the significance of liquid biopsies in a number of cancers, including hepatocellular carcinoma (HCC) [52], pancreatic cancer [53], non-small-cell lung cancer [54], and melanoma [55]. However, the advancement of liquid biopsy research in the field of GC has been comparatively slow, and no review articles of adequate caliber have been published.

In order to detect tumors early, determine prognosis, track tumor burden, predict therapeutic resistance, accurately measure minimal residual disease, and manage cancer in real time for GC patients, liquid biopsies have developed as a new non-invasive technique [56,57]. The term “liquid biopsies” was initially only used to refer to the examination of circulating tumor cells in the blood of cancer patients, but it has since been expanded to also cover the examination of circulating tumor DNA (ctDNA) and circulating noncoding RNAs (ncRNAs) [58].

Large chromosomal alterations, single-nucleotide variations, mutations, aberrant DNA methylation, histone modification, the overexpression or downregulation of microRNAs, and long noncoding RNAs have all been described as major factors implicated in the initiation and progression of GC [59].

**Figure 2:**
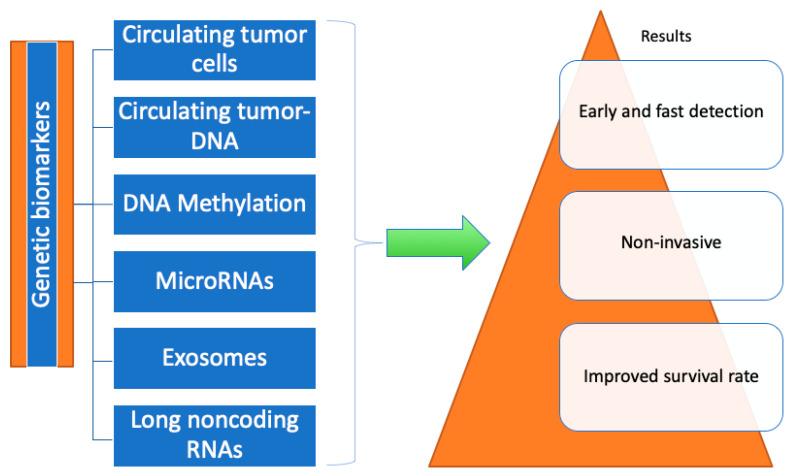
Genetic biomarkers for early cancer diagnosis.

The molecular pathogenesis of GC has recently been better understood thanks to recent developments in next-generation sequencing, which has led to the discovery of potential biomarkers for EGC diagnosis. Circulating tumor DNA, circulating tumor cells, DNA methylation, microRNAs, exosomes, and long noncoding RNAs (as shown in Figure 2) are all promising non-invasive methods for the early diagnosis of GC because tumor cells have the ability to release nucleic acids such as DNA and RNA into the blood. These novel biomarkers provide a greater sensitivity and specificity than the previously referenced protein-based tumor markers (CEA, CA 19-9, etc.). When compared to conventional biomarkers, ctDNA has significantly improved sensitivity and specificity for differentiating patients with GC from healthy people [60,61,62].

A substantial lifetime risk of developing diffuse GC is linked to the cancer syndrome known as hereditary diffuse GC. Genetic testing has become more effective in recent years due to the quick development of data analysis tools and testing technologies. This has made it possible to identify susceptibility genes in individuals, which is important for early cancer detection and the prevention of cancer in members of hereditary cancer-prone families. In those who meet the requirements for genetic testing, screening for CDH1 and CTNNA1 mutations should be carried out. This should be followed by a comprehensive assessment and an interdisciplinary approach to determine the most efficient method of treatment (endoscopic surveillance in comparison to prophylactic gastrectomy).

In conclusion, liquid biopsies have better prospects for repeatable sampling, real-time monitoring, and precision medicine than conventional biopsies. Though challenging, further technological advancement is still necessary to realize the technology’s transformation into clinical practice.

### 4.1. Circulating Tumor Cells

CTCs, which are cells that are generated from solid tumors or metastases and released into the peripheral blood flow, can be thought of as a crucial factor in patients with malignant cancer postoperative recurrence and distant metastasis [63]. Additionally, CTCs might be extremely important in the monitoring and treatment of GC patients with recurrence and metastasis [63]. CTCs have the potential to display particular molecular traits of the primary tumor, which holds great promise for cancer screening [64].

Although many CTCs cannot live in the hostile environment of the vasculature, some of them can [65] thanks to a number of mechanisms. These cells undergo epithelial-to-mesenchymal transition (EMT), which is linked to increased mortality [66], by up- or down-regulating a few surface molecules to weaken cell-to-cell adhesion, enhance plasticity, and move to a secondary site.

CTCs have given researchers the chance to increase sensitivity recently. However, it is more difficult to achieve specificity and sensitivity in GC because CTCs are uncommon in the circulatory system and have much greater heterogeneity [66]. 

The use of CTCs as prognostic biomarkers in various tumors has drawn a lot of attention. The number of CTCs is counted initially. According to reports, CTCs can be used to forecast survival rates and keep track of recurrence after surgery. For instance, Pierga et al. demonstrated that patients with breast cancer who had more than one CTC per 7.5 mL of blood experienced shorter progression-free survival (PFS) (*p* < 0.0001) and shorter overall survival (OS) [67].

Given the cancerous nature and tardy diagnosis of GC, the rapid detection of GC is crucial to ensuring that patients receive successful treatment. The results of the few research projects that concentrated on using CTCs as an indicator for GC were quite unexpected. Yoon-Kyung Cho reported on 115 GC individuals and 31 healthy subjects who provided blood samples totaling 7.5 mL, which were used to separate CTCs using a centrifugal microfluidic system. A specificity of 90.3% was achieved, with 97.1% of subjects who had more than two CTCs being GC patients. The sensitivity of this method needs to be improved, as 38% of GC patients had fewer than two CTCs every 7.5 mL [16].

CTCs may rank among the most significant prognostic biomarkers for a variety of cancers, according to numerous studies. Numerous studies have discovered strong associations between CTC counts and patient survival times. Based on the outcomes of seven studies involving 579 individuals with GC from four different countries, CTCs were considerably associated with low overall survival (OS) in people with GC [68]. 

For instance, an examination of 72 patients found that those with more than one CTC in their blood had shorter disease-free survival (DFS) and overall survival (OS) than those without CTCs (*p* = 0.001 and *p* = 0.0007, respectively) [20]. 

Clinicians can choose the best course of treatment according to CTCs’ characteristics, and CTC changes can be indicative of therapy response. The varied response rates to EGFR-targeted treatment in patients with colorectal cancer (CRC) are explained by the significant intra- and inter-patient heterogeneity in mutant EGFR expression, as well as that of additional genetic changes linked to EGFR inhibition, such as KRAS and PIK3CA mutations [69].

Patients who test positive for CTC (CTC+) have shorter progression-free survival and lower disease control rates (DCR) after therapy than patients who test negative for CTC [68].

Nevertheless, other studies demonstrated that a suitable cut-off value for determining therapy response consists of having more than five CTCs present in the blood. Progressive disease (PD) and shorter PFS were very likely to occur in patients with five or more CTCs [70].

Trastuzumab is the first-line treatment for HER2 + GC, which is characterized by an upregulation of the protein HER2 in a subset of GC cases known as HER2+ or HER2-amplified GC [71]. 

When a tissue biopsy is used to make the diagnosis, HER2 + GC may be mistaken for HER2- GC because of tumor heterogeneity. Trastuzumab cannot be administered to patients in a timely and efficient manner when this happens. According to Mishima et al., 3D-IF-FISH is a cutting-edge method that has a higher sensitivity for detecting HER2 + CTCs than a conventional tissue biopsy, and patients who had HER2+ detected via CTC analysis but HER2- detected via tissue biopsy benefited from trastuzumab therapy [72].

In addition, trastuzumab therapy resulted in a decrease in the number of HER2 + CTCs, which rebounded when drug resistance emerged. Therefore, the quantity of HER2 + CTCs is a helpful indicator of how well trastuzumab therapy is working [73]. 

The conclusion that CTC positivity is associated with poor outcomes has been reached by numerous researchers. As a result, their accurate detection and enrichment pose the biggest challenge in clinical application. But because CTCs are so heterogeneous, it is still difficult to use current technology to detect a wide variety of markers. The processes of tumorigenesis and progression must therefore be studied, and better technologies must be created. In conclusion, CTCs are an important target in GC prognosis that requires additional study.

### 4.2. Circulating Tumor-DNA/Cell-Free DNA

Cell-free DNA, or cfDNA, is made up of circulating cell-free mitochondrial DNA, cell-free fetal DNA, and DNA fragments that have been released by cells into the bloodstream. DNA fragments from cancer cells are referred to as ctDNA [74]. Studies have shown that ctDNA can be released either actively or passively by tumor cells going through apoptosis or necrosis, as well as by primary tumor cells, metastatic tumor cells, and CTCs [75].

CfDNA is essentially absent in healthy people. CfDNA will significantly build up in cases of malignant tumors, ongoing inflammation, and excessive cell death. 

Studies have shown that cfDNA and/or ctDNA analyses are more sensitive methods than conventional biopsies or the evaluation of typical malignant biomarkers used for detecting tumor-specific genetic changes. This is because tumor heterogeneity increases treatment effectiveness; for example, the detection rate of FGFR2 amplification was consistently greater with ctDNA analysis than with a tissue biopsy [76].

Additionally, previous research has demonstrated that quantitative cfDNA determination has diagnostic and prognostic value for a variety of tumors [77,78]. On the basis of this, some investigators probed further into the significance of the single-stranded DNA to double-stranded DNA ratio in circulating cfDNA for GC diagnosis. Their study demonstrated that the ratio of ssDNA to dsDNA in GC individuals was notably lower than that in healthy individuals (*p* < 0.0001), and this ratio had noticeably higher diagnostic specificity and sensitivity than the level of ssDNA or dsDNA alone, with the AUC of this ratio being 0.930 (95%CI: 0.889–0.960), its sensitivity being 83.96%, and its specificity being 94.07%. However, the research also has flaws, including an inadequate sample size, a single tumor type, and a narrow range of ratio fluctuation [19].

Moreover, a study in GC patients receiving PD-1 antibody immunotherapy demonstrated a correlation between declining cfDNA and high immunotherapy responsiveness. The PFS of immunotherapy may be impacted by the baseline cfDNA mutation status of TGFBR2, RHOA, and PREX2 [79].

Since ctDNA and cfDNA have a short half-life and can reflect tumor status almost instantly, they are potential prognostic biomarkers for GC patients [80]. Every GC subject with detectable ctDNA experienced relapse after surgery, and shorter disease-free survival (DFS) and overall survival (OS) were seen in these subjects [81]. Additionally, RFS and OS were worse in GC patients who had a high level of long-fragment cell-free DNA following curative surgery [82].

#### Cell-Free DNA in Comparison to Traditional Biomarkers

Although tumor-associated antigens, such as CEA, CA72-4, and CA50, found on the surface of GC cells can be used as markers for early GC screening, their limitations include high rates of false positive and negative results, poor specificity, and low sensitivity. As a result, a mixed detection system is required [83], and scientists have worked tirelessly to identify a tumor marker that is minimally traumatic, sensitive, trustworthy, and specific to GCs in order to advance early screening.

In their in vivo study using CD1 mice, Czeiger et al. [84] discovered a correlation between the serum CFD concentration and tumor size that was positive. These findings demonstrate that an excessive serum CFD expression may be a sign of disease progression and a poor prognosis. 

The serum CFD concentration in initial (stage I) GC individuals was substantially higher than that in healthy participants, according to an additional analysis of serum CFD levels and clinical TNM staging, but there was no substantial rise in serum CEA, CA19-9, or CA50 levels, indicating that the elevation of serum CFD is an earlier event in GC carcinogenesis than the classical tumor biomarkers, and serum CFD detection may be of major relevance in early GC.

In conclusion, the ease of use and high sensitivity of the branched DNA method for serum CFD level detection make it a potential auxiliary tool. In comparison to serum CEA, CA19-9, CA72-4, and CA50, serum CFD is a more valuable biomarker for GC individuals and a better assistant biomarker for GC early screening.

### 4.3. DNA Methylation

The oncogenesis and development of GC are significantly influenced by epigenetic dysregulations, including DNA methylation, histone post-translational modifications, chromatin remodeling, and noncoding RNAs, according to numerous studies. DNA methylation is the foremost and most thoroughly studied epigenetic modification among those mentioned [85].

DNA methylation is carried out by DNMT1, DNMT3A, and DNMT3B, three different types of DNA methyltransferases. While DNMT1 keeps symmetrically methylated CpGs methylated during DNA duplication, DNMT3A and DNMT3B are primarily ab initio methyltransferases [86,87]. 

In addition to helping with the early detection of GC, the methylation of cfDNA or ctDNA is quickly evolving into a crucial tool for forecasting patient outcomes. For instance, the methylation of PCDH10, RASSF1A, XAF1, SOX17, and WIF-1 in cfDNA or ctDNA has demonstrated great promise [88,89,90].

The hypermethylation of these genes is typically associated with unfavorable outcomes, including relapse, a poor response to treatment, and a shorter survival time. TIMP-3 methylation was found to be highly correlated with peritoneal metastasis and TNM stage and to be upregulated in GC tissue. In peritoneal washes and serum samples taken prior to surgery, it was discovered that TIMP-3 was methylated in about half of GC patients. The TIMP-3 methylation levels in body fluids were associated with shorter disease-free survival in patients [91].

### 4.4. MicroRNAs

MicroRNAs (miRNAs), which are easily detectable, relatively stable in biological fluids, and exhibit high sensitivity, are becoming more and more popular as potential biomarkers that can be detected in liquid biopsies [92]. 

An endogenous RNA with about 22 nucleotides is called an miRNA. A hot topic in molecular research, miRNA is characterized by resistance to RNase degradation and a high stability in biological materials [93].

MiRNAs have been linked to cell proliferation, differentiation, migration, and invasion in previous research [94]. The relationship between serum miR-25 levels and diagnostic foresight was studied by Kong et al. The findings demonstrated that miR-25 enhances both the sensitivity and specificity of GC screening [18]. Yu et al. discovered that members of the miR-200 family increased in EGC [95] and aided in the early detection of GC.

Over the past ten years, miRNAs have attracted a lot of scientific attention. They are a distinct subclass of ncRNAs that play a crucial role in a variety of biological processes. Through the post-transcriptional regulation of gene expression, miRNAs are involved in the regulation of various molecular pathways, such as cell differentiation, cell cycle progression, and apoptosis [96]. Through mRNA targets that encode tumor suppressor genes or oncogenes, deregulated miRNAs can affect the development of cancer [97]. MiRNAs have a number of characteristics because of their distinctive biogenesis, which makes them a desirable group of molecules in the field of biomarker research. MiRNAs can be readily and consistently extracted from a variety of biological materials, including tissues, blood, feces, saliva, ascites, and even blocks that have been embedded in paraffin [98,99].

These small noncoding RNAs have been found to be dysregulated in preneoplastic conditions like early gastric dysplasia, intestinal metaplasia, and atrophic gastritis [100]. With a positive predictive value of up to 90%, certain miRNAs, including miR-21 and miR-376c, have been found to be upregulated in the blood of people with early-stage GC [100].

MiRNA changes are thought to manifest early in the chain of preneoplastic events. For instance, differential miRNA expression has been found in individuals with *Helicobacter pylori* infection, and miR-155 and miR-223 expression in the antrum and corpus mucosa both gradually increased in correlation with Correa’s cascade [98].

After demonstrating the altered miRNA levels in tumor tissues, numerous research teams examined the potential of miRNAs as non-invasive biomarkers in a range of specimens. The expression of miRNAs appears to be measurable in all body fluids (breast milk, urine, synovial fluids, etc.), and it may reflect a normal condition or be linked to pathophysiological changes, thereby supporting their use as biomarkers [101].

More intriguingly, by contrasting malignant tissues with normal tissues, a first-passage observational study found that there were 14 upregulated miRNAs and 8 downregulated miRNAs. Gastric carcinogenesis may be influenced by four miRNAs: miR-31-3p up, miR-6736-3p up, miR-3065-5p down, and miR-3921 down. Only ten samples were included in the study, so it was unable to determine the molecular mechanisms [102].

Single-nucleotide polymorphisms (SNPs) in the miRNA gene are a new area of study for miRNAs. In the Chinese Han population, Song et al. looked at the genotype and allele frequencies of four SNPs in the miRNA machinery genes (GEMIN4, DROSHA, DICER, and AGO1) in GC individuals and healthy subjects. The susceptibility to GC, DICER, GEMIN4, an advanced stage of GC, GEMIN4, AGO1, and the lymphatic metastasis of GC were found to be significantly correlated with rs3742330 (DICER) and rs7813 (GEMIN4) [15]. Additionally, the miR-627 SNP rs2620381 may play a role in the pathogenesis of GC [103].

MiRNAs could be a new class of sensitive and specific GC biomarkers that are non-invasive. The current research is still subject to some restrictions, though. First, the experimental results are quite biased due to the small sample size. Second, it is not clear whether circulating miRNAs can reveal the type of cancer and information about the biological behavior of the primary tumor due to the high degree of heterogeneity in GC. Further research is required to determine the benefits of early GC diagnosis and prognosis. Third, different signaling pathways and miRNAs may be the targets of an miRNA. Also unknown is the intricate molecular mechanism. Fourth, the extraction, measurement, and verification of miRNAs are not subject to a single standard. A promising direction is to use miRNAs developed in the lab for clinical transformation.

### 4.5. Exosomes

In the last ten years, exosomes have received considerable interest in scientific research.

Exosomes, which can also be found in blood, urine, cerebrospinal fluid, and other bodily fluids, are small (30–140 nm) membrane-bound EVs that are fusion-released into the extracellular environment after being produced by wide multivesicular bodies. Exosomes can be produced by a wide variety of cell types, including epithelial, hematopoietic, neuronal, fibroblastic, adipocyte, and tumor cells. Exosomes play a key role in the regulation of both physiological and pathological processes [104].

Exosomes may hold great promise for use as new biomarkers in liquid biopsies, according to a growing body of research. Exosomes have also been looked into for their potential to act as prognostic and diagnostic biomarkers in a number of cancers [105].

Exosomes have been linked to GC tumorigenesis, metastasis, angiogenesis, immune evasion, and drug resistance, according to related research [106].

According to a recent study, exosomal circSHKBP1 is upregulated in GC patients and promotes GC cell proliferation, migration, and invasion by acting as a sponge for miR-582-3p, which upregulates vascular endothelial growth factor (VEGF) and Hu-antigen R (HUR) while inhibiting the degradation of heat shock protein 90 (HSP90). The serum exosomal circSHKBP1 target of liquid biopsies may be useful for GC diagnosis and prognosis [107].

Based on a study by Wang et al., the exosomal delivery of miR155-5p by a paclitaxel-resistant GC cell line can cause chemoresistance in paclitaxel-sensitive cells. Their findings imply that miR155-5p targeting may be a promising treatment for GC chemoresistance [108].

### 4.6. Long Noncoding RNAs

The regulation of cell proliferation, apoptosis, differentiation, and metastasis by long noncoding RNAs (lncRNAs) has been linked to a number of diseases, including tumorigenesis [109]. LncRNAs play a role in the transcriptional, posttranscriptional, and epigenetic aspects of tumorigenesis. LncRNAs can be categorized into sense, antisense, bidirectional, intergenic, and intronic lncRNAs depending on their genomic position related to the protein-encoding gene [110]. The function of lncRNA in the genome is directly influenced by its location. While antisense lncRNAs bind to complementary gene mRNA to prevent mRNA from being degraded by RNase, intergenic lncRNAs control the expression of upstream and downstream genes [111].

The use of tissue or blood lncRNAs as biomarkers for cancer diagnosis has been reported in numerous studies. Because of the specific expression and regulatory differences that they exhibit in various cancers, lncRNAs have a wide range of potential applications as molecular biomarkers. For instance, highly upregulated lncRNA in liver cancer (HULC), is significant for the diagnosis of liver cancer and the identification of hepatic metastasis in colorectal cancer [112], and lncRNA prostate cancer-associated 3 (PCA3) is used in the diagnosis of prostate cancer [113].

Since Wu et al. reported a favorable correlation between H19 overexpression and GC in 1997 [114], increasing research has concentrated on the connection between lncRNA expression and GC risk. LncRNAs have been categorized as tumor suppressors and oncogenic molecules based on their dysregulated expression levels.

The value of lncRNA expression in GC risk evaluation, however, is unclear because some studies have produced contradictory results. In the case of LINC00982, for instance, Fei et al. reported that it was upregulated in GC tissue [115], but Zheng et al. discovered that this structure was downregulated and functioned as a tumor suppressor, and that its excessive expression would impair the proliferative, migratory, and invasive properties of GC cells [116].

According to reports, the long noncoding RNA H19 inhibits apoptosis and promotes GC cell growth [117]. Plasma H19 levels differed between early-stage GCs and non-GCs with an AUC of 0.877, sensitivity of 86%, and specificity of 80% [118], and plasma H19 levels were higher in GCs than in non-GCs [118,119,120]. Additionally, postoperative plasma H19 levels in GC patients were lower than preoperative levels [79]. Consequently, the plasma H19 level may serve as a biomarker to identify GC in its early stages.

Despite the fact that numerous studies on lncRNA profiles are in progress, lncRNAs have the potential to be an important biomarker in the development, spread, and prognosis of GC. Furthermore, the biological behavior of GC cells, such as proliferation, invasion, metastasis, and drug resistance, is predicted by the expression levels and intricate signal pathways. As a result, lncRNAs are anticipated to develop into a potent tool for targeted therapy, though the full mechanism is still unknown. Additionally, the abundance, simplicity of degradation, and instability of the current measurement render it difficult.

Elevated degrees of heterogeneity and a lack of preliminary symptoms are features of GC. Therefore, for a positive outcome, GC must be diagnosed quickly and early. Today, using biomarkers to diagnose GC early and effectively has become a new diagnostic option. According to reports, GC biomarkers have excellent potential for early tumor detection, prognosis assessment, monitoring tumor burden, predicting drug resistance, and providing current information on treatments.

By identifying circulating tumor markers in bodily fluids, liquid biopsies, a non-invasive method, are anticipated to achieve repeated sampling, real-time monitoring, and precise treatment. In general, the potential biomarkers found in liquid biopsies, such as CTCs, lncRNAs, cfDNA, miRNAs, and exosomes, offer a wealth of data regarding the prognosis and early prediction of GC patients.

It is also attainable to use the discovery of precise biomarkers closely linked with GC development for diagnosis and treatment. This review will eventually aid in the identification of reliable biomarkers in clinical GC patient care for the most beneficial GC prevention and treatment.

### 4.7. CDH1 (E-Cadherin)

Hereditary diffuse gastric cancer (HDGC) is a cancer syndrome linked to a substantial lifetime risk of both lobular breast cancer and diffuse gastric cancer (DGC), a disease with a poor outlook and a delayed clinical presentation. Germline pathogenic variations in the E-cadherin gene (CDH1), which are inherited in an autosomal dominant pattern, are associated with HDGC. The CDH1 gene (cadherin 1) has germline mutations in about 40% of HDGC families. The homophilic transmembrane protein E-cadherin, which is localized to adherens junctions in epithelial tissue and plays a tumor suppressor role, is encoded by the CDH1 gene. For cell–cell adhesion, cell mechanosensitivity, epithelial–mesenchymal transition, and the contact inhibition of cell proliferation in normal cells, E-cadherin expression is essential [121].

Currently, genetic testing based on personal history, family history, a detailed three-generation pedigree evaluation, and other risk models is used to identify individuals at risk of HDGC syndrome due to the autosomal-dominant inheritance and the high penetrance of CDH1 germline mutations. There are two primary clinical methods for genetic testing: multiplex genetic panel testing (MGPT) and single-gene testing. DNA obtained from blood or buccal samples can be used for both tests, with the exception of individuals who have recently been diagnosed with hematologic malignancy or who have undergone allogenic bone marrow transplantation. The ideal tissue sample for these patients is fibroblast culture DNA.

Early genetic counseling and the identification of CDH1 mutations in carriers who are asymptomatic have been shown to improve survival in HDGC, according to retrospective data. However, Xicola et al.’s study, which involved 476 relatives and 113 CDH1 pathogenic variant probands, showed that the GC risk is low in unselected CDH1 pathogenic variant carrier families that do not meet HDGC requirements [122]. Moreover, in families pre-selected for HDGC criteria, the age at first diagnosis was higher than previously documented. A significant percentage of families with breast cancer do not have any GC [122].

A multimethod technique for CDH1 testing utilizing a range of molecular techniques, such as DNA sequencing, MPLA, single-nucleotide primer extension, bisulfite sequencing, reverse-transcription PCR, and bioinformatics tools, is supported by a study published by Molinaro et al. It illustrates that, in order to improve the rate at which pathogenic mutations are discovered, both DNA and RNA analyses are necessary. This approach has the potential to decrease the number of patients lacking a definitive molecular diagnosis [123].

The last few years have seen the rapid development of technologies for genetic testing, as well as that of data analysis tools. This has made genetic testing more effective and facilitated the identification of HDGC susceptibility genes.

Nonetheless, a multidisciplinary team comprising gastroenterologists, genetic counselors, medical geneticists, pathologists, and oncological psychologists is crucial in determining the proper clinical management and decision making. Unaffected carriers from HDGC families must make difficult decisions due to the variable penetrance of the CDH1 or CTNNA1 mutation. The best way to support them is through education and interactions with counseling from a knowledgeable interdisciplinary team [124].

## 5. Metabolic Biomarkers of GC

The most recent addition to the “omics” family of disciplines, which includes genomics, transcriptomics, and proteomics, is metabolomics. A system’s flow of biological information from DNA to RNA to protein to metabolites is described by the central tenet of molecular biology. At various points in this dogma, various “omics” interventions are used to obtain a glimpse of how cells, tissues, and organisms function internally. The totality of an organism’s low-molecular-weight (1500 Daltons) metabolites make up its metabolome [125].

A cell needs metabolites to maintain itself, grow, and function normally. Mapping the metabolomic profile offers a comprehensive view of the organism at a particular time and under a particular set of circumstances. A small genomic change can be multiplied many times at the metabolite level for any given disease state and quantified. Researchers can measure metabolites in biological samples like tissues, urine, saliva, and blood plasma to pinpoint particular metabolic pathways. Previous research has shown that the metabolic processes of cancer cells differ noticeably from those of healthy cells. The metabolomic profile can be used to distinguish specific cancer biomarkers and offer clues for an early diagnosis.

Any biofluid, such as serum, urine, or fecal water, is suitable for metabolic studies. Given that each sample has both advantages and disadvantages, as shown in Table 2, the one chosen should be the most appropriate for addressing the clinical need.

Blood and other biofluids can be obtained with little to no invasiveness, making them ideal study samples. These biofluids’ profiles can be traced to their genetic roots to reveal disease pathways. Metabolites reflect cellular conditions at the time of sampling and can be thought of as “endpoint markers” for disease because they are “downstream” entities compared to genes. Nuclear magnetic resonance spectroscopy (NMR), mass spectrometry (MS), and liquid and gas chromatography are currently used to analyze the metabolome.

Dysregulated metabolism, during which cancer cells exhibit increased glucose uptake and lactate production, is one of the characteristics of cancer. The “Warburg effect” refers to this process, but it is unclear how and why malignant cells alter their metabolic state. Cellular growth has been linked to a number of metabolic changes related to cancer; in fact, the biosynthesis of lipids, proteins, and nucleic acids is necessary for the development and survival of tumors. Most often, changes in the expression, activity, or flow of the primary metabolic pathways result from the expression of oncogenes or the loss of tumor suppressors. Various glucose and glutamine (Gln) metabolism elements have been identified as crucial cancer metabolism regulators. Given the significance of metabolic changes in cancer development and prognosis, metabolomics represents a fundamental area of omics research because it can be used to assess changes in primary metabolites [126].

This metabolic reprogramming may aid in the early detection of tumors, and biological fluids may provide valuable information, obviating the need for invasive screening. An NMR analysis can be used to find potential biomarkers linked to cancer risk, presence, and prognosis in urine and blood, two readily accessible matrices [127].

The utilization of metabolomics-based approaches to study cancer metabolism is becoming more and more popular. NMR and mass spectrometry (MS) are the two main instrumental metabolomic methods. These two techniques’ benefits are fundamentally different from one another. For metabolomics research, the MS platform offers sensitivity and selectivity, whereas NMR offers extremely high reproducibility, is quantitative, and only needs a few steps for sample preparation, preventing the need for separation or derivatization [128].

Therefore, metabolomics performed using readily available biofluids may have an impact on the conventional clinical practice of cancer diagnosis, prognosis, and risk assessment.

### 5.1. Serum Biomarkers

The presence of tumor markers like the carbohydrate antigen 19-9 and the carcinoembryonic antigen can be detected in serum samples, but due to the possibility of false positive results from other non-neoplastic conditions, these tests have good sensitivity but poor specificity, as mentioned above. Therefore, it is important to create alternative screening tools that enhance early detection and precise cancer classification. Blood metabolomics has shown promise in aiding GC diagnosis.

#### Differential Diagnosis between CG vs. GC Using Metabolomics

Serum markers can reveal patients’ systemic metabolic dysregulation. The best serum biomarkers for clinical use are non-invasive and affordable. In order to find significantly altered pathways and different metabolites, untargeted metabolomics has been used to analyze the serum metabolomic profiles of patients with CG or GC. Fasting lipid profiles have also been examined. The candidate biomarkers can be utilized to diagnose CG and GC, and this study may shed light on the pathogenesis of GC.

The biochemical basis for tumorigenicity and malignancy is often a fundamentally altered cellular metabolism, which is frequently seen in cancer cells. Therefore, the pharmaceutical industry and clinical research have recently become very interested in the topic of cancer metabolism.

According to one study, the transition from CG to GC may be influenced by lipid metabolism. Following a fast of between 8 and 14 h, serum samples were taken in the morning and placed into 5 mL tubes (BD Vacutainer^®^ SST II Advance tubes). The samples were either stored at 80 °C until analysis or centrifuged at 3000 rpm for 15 min before being analyzed within 4 h [21].

Lipid metabolism involved almost all metabolic pathways, including sphingolipid, glycerophospholipid, and arachidonic acid metabolism.

In contrast to healthy individuals or CG patients, patients with GC had lower serum levels of total cholesterol (TC), high-density lipoprotein cholesterol (HDL-C), and apolipoprotein A1 (ApoA1) [129].

The serum levels of these metabolites were significantly lower in GC patients than in CG patients, suggesting a potential link between lower levels of TC, HDL-C, and ApoA1 and the development of CG into GC. According to the outcomes of other studies [130,131], a decrease in the lipid profile in cancer individuals may be caused by an increase in the utilization of lipids by neoplastic cells during membrane biogenesis. The development of CG to GC may be linked to lipid metabolism, according to the findings of an untargeted metabolomic analysis and the fasting lipid profile. The precancerous cascade of the multi-step process that leads to GC begins with CG [132,133]. However, it is still unknown how gastritis begins and develops into GC.

Current thinking holds that an altered lipid metabolism is a defining feature of many malignancies, and lipids have generated growing interest as potential biomarkers in a variety of clinical conditions. Therefore, we looked into the serum diagnostic capabilities of these vastly different metabolites. The findings showed that hexadecasphinganine and linoleamide can be used as candidate biomarkers for GC, while N-Hydroxy arachidonoyl amine and linoleamide can be used as candidate biomarkers for CG. The combination of these biomarkers can improve diagnostic precision for differentiating between CG and GC. As a result, single biomarkers can diagnose CG or GC in contrast to healthy subjects with high sensitivity and specificity. However, the results obtained by combining the three indicators for the differential diagnosis of CG compared to GC enhanced the ability to discriminate provided by diagnostic procedures based on single markers. Hexadecasphinganine, linoleamide, and N-hydroxy arachidonoyl amine are three lipid compounds with high potential diagnostic value that could be considered potential biomarkers for establishing a diagnosis of CG or GC. Linoleamide, a type of fatty acid, also has anti-inflammatory properties, for instance [134]. Although these substances have been studied in other diseases, it is unknown what role these three metabolites play in the pathophysiology of GC [129].

### 5.2. Urinary Biomarkers

The search for biomarkers has increased the interest in urine, a desirable biofluid. When comparing urine to plasma and serum, which are more difficult to obtain, it was observed that urine is a highly coveted biospecimen for biomarker analyses [135].

The use of urinary biomarkers in tumors of the excretory or genitourinary cancer system, including bladder cancer, prostate cancer, and upper urinary tract urothelial carcinoma, has developed over time, and some urinary biomarkers have already finished the confirmatory stages of clinical use [136].

Significant advancements have been made in GC screening for urinary biomarkers, particularly for early-stage tumors. However, age, sex, diet, hormonal status, and exercise habits can all have an impact on urine [137]. Therefore, additional research is needed to confirm the potential biomarkers’ universal applicability, and the standardization of experimental protocols is necessary.

Additionally, recent advances in mass spectrometry, nuclear magnetic resonance, gas and liquid chromatography (LC), and capillary electrophoresis (CE) have improved reproducibility and metabolome coverage in urinary metabolomics [126].

Surface-enhanced laser desorption ionization MS, tandem MS (MS/MS), LC-MS, CE-MS, array technology, and surface-enhanced laser desorption ionization MS have all been used for proteomic studies to analyze urine and find biomarkers [138].

Numerous investigations have looked at urinary metabolites for GC detection. GC diagnostic biomarkers include oxidative nucleic acid metabolites, amino acids, and bile acids. Gas chromatography coupled to mass spectrometry (GC-MS) was used in a previous study to analyze the metabolites in 293 urine samples. The results showed that the levels of 10 amino acids in the urine (valine, alanine, proline, tryptophan, isoleucine, serine, threonine, tyrosine, methionine, and glycine) were significantly higher in GC patients and had a diagnostic range of 0.693 to 0.823 [139].

In addition, Chan et al. discovered higher urinary alanine concentrations in GC patients than in healthy participants. Additionally, they developed a diagnostic model for GC based on alanine, 2-hydroxyisobutyrate (2-HIB), and 3-indoxylsulfate (3-IS), which has an AUC of the corresponding receiver operating characteristic curve of 0.95, a specificity of 80%, and a sensitivity of 95% [13].

Despite the positive outcomes of these mentioned retrospective studies, new NMR metabolomics-based prospective studies should be carried out. Urinary metabolomics could be a good non-invasive alternative to determine tumor-associated perturbations. The latter could show the value of NMR metabolomics for the detection of GI cancers using urine samples if validated with independent and wider cohorts. In fact, a urine metabolomics analysis is a simple method that can be used for mass population screening. The variability of the samples, which is caused by a variety of host (such as dietary and lifestyle habits) and environmental factors, as well as the pathophysiological status of the patient, is the urine metabolomics profile’s biggest drawback in clinics. Controlling these variability factors during the experimental design should receive more focus.

### 5.3. Fecal Biomarkers

Even though fecal metabolomics is becoming more and more popular, there is not yet a standardized way to gather, prepare, and analyze fecal samples. The fact that this type of mixture is a semi-solid combination of endogenous and exogenous components, which necessitates a quite complex sample preparation for metabolomics analyses, exacerbates this lack of standardization. In addition, unlike urine, serum, plasma, cerebrospinal fluid, and saliva biofluids, a fecal metabolite analysis has not often been the subject of a systematic review or study.

To calculate the concentration of metabolites in fecal extracts in colorectal cancer (CRC), three studies considered a metabolic analysis with NMR. Fecal water extracts are rich in small metabolites like lactate, glucose, and amino acids, according to the results of the first study on CRC by Monleón et al. [140], who used 1H-NMR on a small cohort of 11 controls and 21 CRC patients. Due to the lack of a nutrition control, the spectra showed high levels of variability. However, a multivariate analysis revealed important distinctions between the two categories. Similar outcomes were found in two separate studies by Lin et al. [141,142].

In Lin et al.’s study, 1H NMR spectroscopy in conjunction with an orthogonal partial least squares-discriminant analysis (OPLS-DA) provided compelling evidence that the fecal metabolic profiles of CRC patients were different early on from those of healthy controls. Their understanding of the colonic molecular pathogenesis underlying disease processes may be expanded by the altered fecal metabolites, which may have revealed disruption of the normal bacterial ecology, the malabsorption of nutrients, increased glycolysis, and glutaminolysis. These findings may be correlated with the onset and progression of colorectal cancer [141].

Acetate can be converted to acetyl-CoA for lipid biosynthesis, and it serves as a compound for endogenous cholesterol. These findings support the notion that the switch to lipogenesis is a classic modification of cancer metabolism. The acetate levels in CRC feces and the glucose and myo-inositol levels in colorectal cancer tissues were found to be related by Lin and his team. Significant reductions in the levels of acetate in feces and in CRC tissues’ glucose and myo-inositol may be signs that the cancer cells are using more energy to grow. Leucine levels have been found to be higher in the feces of CRC individuals than in those of healthy subjects; this could be because of epithelium inflammation, which impairs nutrient absorption [143]. However, because there is no dietary monitoring in patients, the amino acid metabolic profile is frequently very diverse.

Fecal water samples may prove to be intriguing and affordable biofluids to investigate for future applications, despite the limited research using stools for a metabolic NMR analysis.

The relative abundance of metabolites differs significantly not only between GC and controls but also between different stages of cancer. One study’s upregulated metabolites might be downregulated in another. The analytical method (GC/MS/NMR), sample selection (blood/urine/tissue), or subject type (animal/human) may all be to blame for this.

## 6. Future Perspectives

The mainstay of treatment and a significant factor in improving patient quality of life is the early endoscopic discovery and removal of superficial gastrointestinal (GI) neoplasms. The most effective methods for identifying early superficial neoplasms are routine esophagogastroduodenoscopy (EGD) and colonoscopy. The 21st century has largely eliminated the need for surgical intervention thanks to advancements in resection techniques like conventional snare polypectomy, endoscopic mucosal resection, and endoscopic submucosal dissection. As a result, the significance of the early detection of superficial neoplasms keeps growing.

Dye-based image-enhanced endoscopy (d-IEE), which includes chromoendoscopy using indigo carmine, is helpful because it highlights mucosal irregularities to improve endoscopic detection [144]. However, it is not efficient to spray the whole GI tract, so indigo carmine must be sprayed through the working channel. The rate of endoscopic diagnosis has significantly increased over the past 20 years thanks to the development and adoption of equipment-based image-enhanced endoscopy (e-IEE) modalities like narrowband imaging, blue laser imaging (BLI), and flexible spectral image color enhancement (FICE).

For a thorough characterization of identified lesions, NBI and BLI with magnification are helpful. These techniques are helpful for finding lesions in the esophagus and other constrictive regions of the GI tract. However, because they lack the light intensity to illuminate a wide lumen, they are ineffective for locating lesions in the stomach and colon. In comparison to standard BLI, BLI-bright offers brighter images and a narrowband analysis, even in wide lumen organs like the stomach and colon. When viewed from a distance, BLI-bright more accurately highlights mucosal vessels and structures than white light imaging (WLI). Real-time EGC detection rates using BLI were found to be significantly higher than those using WLI in a Japanese prospective study [145].

FICE, in contrast to NBI, has a sufficient intensity of light to illuminate a large lumen. As a result, it can sometimes be used to identify early-stage tumors, but it is insufficient for displaying the specifics of the mucosal structure and blood vessels. At a distance, FICE is unable to differentiate between neoplasms and intestinal metaplasia in the stomach. The use of e-IEE for the earliest identification of superficial neoplasms during regular endoscopic evaluations is not supported by enough evidence. To reduce the mortality from GI tract malignancies, it is imperative that e-IEE develops to improve the recognition of superficial GI neoplasms at a distance.

Clinicians frequently come across situations where it is difficult to diagnose GC or the presence of a *Helicobacter pylori* infection using traditional white light imaging (WLI). Machine learning models and image-enhanced endoscopy (IEE) through linked color imaging (LCI) are two techniques created to make such findings more widely achievable.

### 6.1. Linked Color Imaging

A recently created IEE modality called linked color imaging (LCI) is founded on the BLI technique and uses digital image processing. (LESEREO endoscopic system; Fujifilm Co., Tokyo, Japan). LCI combines a laser light source with two wavelengths: a 410 nm short-wavelength narrowband laser and a 450 nm white light laser. The L*a*b* color space, where the a*-b* plane stands for hue and saturation and the L*-axis for brightness, is used to explain the effectiveness of LCI. Signal processing emphasizes the color of the gastric mucosa by enhancing the red (+ a*) and yellow (+ b*) directions; as a result, a large proportion of EGCs are seen as light colors with elevated b* values in the reddish gastric mucosa. Contrarily, an area of intestinal metaplasia is visible as a purple color and a low b* value. By maintaining the pertinent L* axis component, brightness is guaranteed. Thus, LCI maintains adequate brightness for mid- to long-range monitoring in gastric screening while differentiating mucosal colors that are crucial for EGC diagnosis.

The definition of suspicious lesions was standardized because endoscopists typically identify EGCs based on suspicious lesions, which were defined as the presence of mucosal surface and color changes [146]. In a prior study [147], a diagnosis of EGC was established via WLI observation, and the lesions’ distinct red and pale colors were then classified as either differentiated or undifferentiated types. Beyond the presence of a clearly defined border, previous research advocated for a yellow-red color in the suspicious lesion to be included in the definition of EGC by using LCI. Lesions were then classified as differentiated or undifferentiated depending on the presence of purple in the peripheral mucosa.

#### 6.1.1. LCI Used for Diagnosis of HP+ Gastritis and Premalignant Lesions

Chronic gastritis, major atrophy, and intestinal metaplasia brought on by *Helicobacter pylori* (HP) are all generally regarded as risk factors for developing GC [148].

WLI can identify endoscopic indicators of intestinal metaplasia such as ash-colored nodular changes. The diagnosis of gastrointestinal metaplasia in chronic gastritis is challenging, despite the high risk of GC [149]. According to the available data, gastrointestinal metaplasia manifests as green mucosa with inconsistent distributions on BLI and violet (lavender color) mucosa on LCI. BLI may be utilized for detecting green mucosa, which highly indicates intestinal metaplasia if it cannot be determined on LCI whether the mucosa is faded purple or another color [149,150].

On LCI, intestinal metaplasia is clearly visible as lavender, whereas the eradicated HP-negative mucosa is easily identifiable as apricot. Disperse redness of the gastric mucosa of the gastric body, an usual sign of an ongoing Hp infection, is observed precisely as deep red (crimson) in color. It has been noted that the rate of identification of LCI for intestinal metaplasia is considerably greater than that of WLI due to these distinct color differences [151].

According to reports, LCI can be suitable to outline the borders of gastric atrophic and nonatrophic mucosa by comparing the colors of the two [152]. When using LCI, the variation in color at the atrophic border is notably greater than when using WLI. A few prospective studies claimed that gastric intestinal metaplasia could be identified as purple (lavender color) using LCI and as a white flat/elevated lesion using WLI. This condition is thought to be a risk factor for intestinal-type GC. When compared to WLI, the diagnostic accuracy of a biopsy was improved when LCI was used [153]. LCI is therefore helpful in identifying stomach premalignant lesions.

#### 6.1.2. LCI Used for Detection of EGC Lesions

Regular observation using WLI alone frequently misses EGC hiding behind chronic mucosal inflammation. The lumen of the stomach is wide, unlike the esophagus, and it is too large for NBI and BLI to adequately illuminate. This makes these e-IEE modalities useless for standard EGD. LCI can illuminate a large lumen with enough light intensity to make it easier to see superficial neoplastic lesions in the stomach. Because their weak light intensity produces dark images, NBI and BLI were not frequently utilized for screening endoscopy for early malignancies of the stomach prior to the development of LCI.

Even for skilled endoscopists, it can be challenging to differentiate between neoplastic lesions and the inflammatory mucosa brought on by a persistent HP infection. Intestinal metaplasia typically surrounds the majority of GCs. Because both gastric neoplasia and metaplasia have similar characteristics using WLI, the diagnosis of GC is challenging when widely spreading intestinal metaplasia is present. Although the development of magnifying endoscopy in recent years has helped to resolve this issue, gastroenterologists still occasionally struggle to see all of the numerous gastric lesions under magnification.

In LCI, EGC has an orange-red appearance and is encircled by intestinal metaplasia and purple mucosa. In EGC, the microvasculature in the superficial layer of the mucosa can absorb violet light, and unlike inflammatory mucosa, GC appears orange-red using LCI.

LCI typically shows an orange-red appearance for GC [154]. We first revealed that LCI is superior to WLI because it clearly distinguishes between a flat GC (orange) and the nearby intestinal metaplasia (purple), which was challenging to find using WLI [155]. According to a Japanese study, using LCI rather than WLI significantly increased the distinction in color between GC and the adjacent mucosa. When intestinal metaplasia was present around a GC, the color difference was harder to ignore [156].

In cases of initial-stage tumors with adjacent intestinal metaplasia, LCI may therefore be most helpful. In LCI, intestinal metaplasia that surrounds the EGC has an orange-red aspect and is encircled by purple mucosa. In LCI, EGCs are typically orange-red, orange, or orange-white [154].

However, because of its magnified views, BLI outperforms LCI in the identification of odd discoveries in the microstructure and microvascular system of the GC. Therefore, it is crucial to use LCI to find GC (orange) that is encircled by intestinal epithelial metaplasia (purple) and to use magnifying BLI to confirm the diagnosis.

Along with a pre-existing morphological diagnosis, LCI offers significant color distinction between neoplastic and inflammatory lesions. This color differentiation may be significantly influenced by the histological differences in the superficial layer of the mucosa, which includes the depth and density of the capillaries and changes in the glands. Enhancing the color contrast makes it much easier to tell neoplastic lesions apart from non-neoplastic mucosa. LCI can only help with the recognition of lesions in visualized GI mucosa, so we must proceed with caution.

The ability to identify lesions in blind spots, such as those concealed by folds or sharp bends, cannot be improved by LCI. Basic endoscopic instruction and careful examination taking sufficient time are still very important because LCI cannot eliminate the miss rate of all GI lesions. The value of LCI for the distant detection of GI neoplasms is clear despite the paucity of rigorous evidence. The authors advise starting routine EGD and colonoscopies with LCI.

### 6.2. Machine Learning Models

Recent research has shown that innovative techniques like machine learning and big data mining are useful for enhancing disease diagnosis, prediction, biomarker selection, and screening in the medical field [157].

Due to their computational power, the widespread use of artificial intelligence (AI)-based solutions, such as machine learning (ML), can overcome the limitations of invasive diagnostic methods in GC detection and diagnosis. The development of predictive and data analysis models frequently uses machine learning (ML) techniques, which also have the ability to implicitly extract useful data from unstructured datasets [158].

Without explicit programming, ML models can be generated automatically by training data and can be used to draw conclusions or make decisions under uncertain circumstances [159]. ML algorithms can improve prediction accuracy more than conventional statistics methods by capturing multifaceted nonlinear relations in datasets [160,161].

One of the fundamental components of AI is machine learning, which has the ability to predict the future or make decisions on its own without the need for human guidance [162].

The use of AI in the endoscopic diagnosis of gastric tumors extends beyond simple detection to include both characterization and classification. The depth of the wall invasion of GC was determined in endoscopic images by a computer-aided pattern recognition system [27] and a convolutional neural network computer-aided detection (CNN-CAD) system [5]. Patients were also screened using these systems.

In addition, both intestinal metaplasia and mucosal atrophy brought on by chronic gastritis linked to HP may raise the risk of GC [148]. As a result, the early detection and prevention of GC depend greatly on a reliable identification of HP infection. To identify HP infection early and stop GC, a CNN system was created that can identify particular elements of gastric endoscopy images. The maximum sensitivity and specificity of these systems were 88.9% and 87.4%, respectively, and the accuracy ranged from 83.1 to 87.7% [163,164]. The CNN-assisted system performed significantly better than endoscopists in terms of accuracy and efficiency. This demonstrates that AI-assisted HP infection diagnosis is feasible and is anticipated to support and enhance GC early diagnosis.

It should be made clear that pathologists’ depth of knowledge and contextual awareness cannot be replaced by AI; rather, a combination of the two is necessary to best demonstrate the benefits of AI. The efficiency and accuracy of pathologists in diagnosing GC have been improved by artificial intelligence. The impact of false positives and false negatives on individuals is understood by pathologists, who are then able to optimize diagnostic procedures to better meet the needs of specific clinical situations. With AI’s help, physicians’ workloads are significantly reduced, allowing them to focus more on challenging cases [165].

Adapting AI to enhance diagnoses for GC is a worthwhile endeavor despite increasing efforts. How we approach problems with GC may be completely changed by the information that is produced. Even though integration may be laborious and slow, it has the potential to improve treatment plans and visual modalities for diagnosis. It has the potential to develop into an essential tool for doctors, but only if people learn to teach it and adapt it.

## 7. Discussion

Because GC rarely exhibits symptoms in the early stages, early detection is both important and difficult, contributing to the disease’s high mortality rate. In some nations where routine screenings are not conducted, the majority of cases of stomach cancer are not discovered until it is too late and the disease has spread to other tissues.

Globally, EGC has a very good prognosis. The geographic variation in EGC prevalence is significant, and EGCs are rarely ignored in high-prevalence areas with good diagnostic capabilities. Endoscopic mucosal resection and pylorus-preserving gastrectomies are examples of less invasive, function-preserving treatments that are now commonplace in these nations. Along with technological advancements, the criteria for applying conservative treatments are being expanded. EGC should be carefully addressed because it may be fatal despite having a lengthy natural history. The most crucial prognostic factor for EGC that should be taken into account when choosing a treatment and creating a follow-up plan is lymph node metastasis.

The discovery of the idea of EGC has resulted in novel diagnostic and therapeutic advancements that are currently being utilized to improve outcomes for gastrointestinal conditions like GC and other illnesses. Additionally, improvements in the creation of innovative biomarkers (genetic and metabolic markers) and AI (through machine learning models and LCI) hold the promise of a quicker and more accurate diagnosis of GC. These results, along with the superb results of EGC management, support the need for GC screening in the newly high-risk population.

Conventional techniques possess certain limitations, such as the low sensitivity and specificity of classical tumor markers, the radiation-induced toxicity of CT and radiographic scanning, and the inability to visualize early lesions. In terms of endoscopy, it is a procedure that depends on the operator, and sometimes the diagnosis may be delayed due to the challenge of obtaining adequate tissue samples. There are some limitations to this study. The availability of the data regarding metabolic biomarkers was limited. Also, the study protocol was not registered in the public PROSPERO platform, mainly because of the extended waiting time for registration.

## 8. Conclusions

A histological examination of the stomach mucosa is the gold standard for identifying GC in its early stages. However, this method’s invasiveness makes it unsuitable for population screening. In clinical practice, a histopathological diagnosis is very helpful for both the definitive and supportive diagnosis of GC, but it has certain limitations. Through a molecular analysis of the changes in histopathology specimens, we might be able to overcome the limitations of histomorphology-only diagnoses, improving our ability to diagnose early lesions of the stomach, learning more about the grade of malignancy, and identifying patients who are at a higher risk of developing primary cancers. Therefore, in certain instances, genetic and metabolic biomarkers may serve as non-invasive alternatives to a conventional invasive physical biopsy.

EGC has a very good prognosis if the lesion is entirely eradicated through endoscopic resection or surgery. Early diagnosis is crucial because it increases survival rates and allows for earlier treatment; therefore, the identification of novel and specific markers to diagnose and predict GC is imperative.

## Figures and Tables

**Table 1:** Characteristics of the included studies.

	Population	Patients	Sensitivity %	Specificity %	Diagnosis Method	Sample Type	Biomarker
Chan et al. (2016) [13]	Mixed (GC + CG + healthy controls)	43 + 40 + 40	95%	80%	Metabolic	Urine	Multiple
Shichijo et al. (2017) [14]	Mixed (GC + healthy controls)	753 + 1015	81.9%	83.4%	Endoscopic	-	Machine learning models
Song et al. (2017) [15]	Mixed (GC + healthy controls)	628 + 502	Missing data	Missing data	Genetic	Serum	MicroRNA
Hwa Mi Kang et al. (2017) [16]	Mixed (GC + healthy controls)	116 + 31	85.3%	90.3%	Genetic	Serum	Circulating tumor cells
Itoh et al. (2018) [17]	Mixed (HP+ and HP-)	65 + 74	86.7%	86.7%	Endoscopic	-	Machine learning models
Kong et al. (2019) [18]	Mixed (GC + CG + healthy controls)	184 + 56 + 78	67.3–69.4%	80.4–81.0%	Genetic	Serum	MicroRNA
Huang et al. (2020) [19]	Mixed (GC + healthy controls)	106 + 118	83.96%	94.07%	Genetic	Serum	Circulating cell-free DNA
Qian et al. (2021) [20]	GI cancer patients	72	Missing data	Missing data	Genetic	Serum	Circulating tumor cells
Yu et al. (2021) [21]	Mixed (GC + healthy controls)	72 + 51	75.73%	70.36%	Metabolic	Serum	Multiple

GC = gastric cancer, CG = chronic gastritis, GI = gastrointestinal.

**Table 2:** Benefits vs. detriments of biofluids for metabolic studies.

	Advantages	Disadvantages
Blood	Minimal preparation of the sample Provides data on a systemic basis Offers a more stable and well-defined metabolome	Invasive method Requirement for specialized personnel in the collection process
Urine	Non-invasive Minimal preparation of the sample No requirement for specialized personnel in the collection process Obtainable in large quantities upon request	Metabolome variability (strongly impacted by consumption of food and water)
Fecal water	Non-invasive No requirement for specialized personnel in the collection process Obtainable in large quantities upon request In association with the microbiota	Metabolome variability (strongly impacted by consumption of food and water) Sample preparation requires more time

## Data Availability

Data are contained within the article and Supplementary Materials.

## Supplementary Materials

The following supporting information can be downloaded at: https://www.mdpi.com/article/10.3390/diagnostics13243608/s1, PRISMA 2020 Checklist [12].
